# Personalized nutrition: A review of genotype-based nutritional supplementation

**DOI:** 10.3389/fnut.2022.992986

**Published:** 2022-09-09

**Authors:** Feijie Wang, Jianheng Zheng, Junrui Cheng, Hong Zou, Mingfeng Li, Bin Deng, Rong Luo, Feng Wang, Dingqiang Huang, Gang Li, Rao Zhang, Xin Ding, Yuan Li, Jun Du, Yuexin Yang, Juntao Kan

**Affiliations:** ^1^Nutrilite Health Institute, Shanghai, China; ^2^Department of Molecular and Structural Biochemistry, North Carolina State University, Kannapolis, NC, United States; ^3^Sequanta Technologies Co., Ltd, Shanghai, China; ^4^Nutrilite Health Institute, Guangzhou, China; ^5^School of Public Health, Institute of Nutrition and Health, Qingdao University, Qingdao, China; ^6^Chinese Center for Disease Control and Prevention, National Institute for Nutrition and Health, Beijing, China

**Keywords:** personalized nutrition, nutritional supplementation, nutrient, genotype, single nucleotide polymorphisms, nutrigenetics

## Abstract

Nutritional disorders have become a major public health issue, requiring increased targeted approaches. Personalized nutrition adapted to individual needs has garnered dramatic attention as an effective way to improve nutritional balance and maintain health. With the rapidly evolving fields of genomics and nutrigenetics, accumulation of genetic variants has been indicated to alter the effects of nutritional supplementation, suggesting its indispensable role in the genotype-based personalized nutrition. Additionally, the metabolism of nutrients, such as lipids, especially omega-3 polyunsaturated fatty acids, glucose, vitamin A, folic acid, vitamin D, iron, and calcium could be effectively improved with related genetic variants. This review focuses on existing literatures linking critical genetic variants to the nutrient and the ways in which these variants influence the outcomes of certain nutritional supplementations. Although further studies are required in this direction, such evidence provides valuable insights for the guidance of appropriate interventions using genetic information, thus paving the way for the smooth transition of conventional generic approach to genotype-based personalized nutrition.

## Introduction

Nutrition disorders, such as obesity, cardiovascular disease, and diabetes, primarily driven by the unhealthy diet and/or lifestyle, are the leading cause of premature deaths worldwide (1). Adherence to dietary guidelines, with the recommended intake of specific nutrients, is necessary for health maintenance at the population level (2). However, this one-size-fits-all approach does not consider interindividual variations, which result from varying responses to nutrients owing to different genetic predispositions, metabolic phenotypes, and microbial compositions. Therefore, personalized nutrition, which is adapted to individual needs, has been proposed to meet this challenge.

The concept of “personalized” or “precision” has been widely used since the Precision Medicine Initiative was launched in the United States in 2015 (3). Similar to precision medicine, personalized nutrition refers to the use of unique information about an individual to tailor nutritional interventions, including advice, products, and services, to assist them to gain improved health benefits than those derived using generic, population-based approaches. With the advances in “omics,” such as genomic, transcriptomic, proteomic, metabolomics, microbiome and data technology, personalized nutrition has gradually become a reality (4, 5). Among them, genomics has evolved to the post-genomic era, and genomic information has been widely used to tailor personalized nutrition for certain nutritional supplementations, yielding the interdisciplinary science called nutrigenetics. According to the International Society of Nutrigenetics/Nutrigenomics (ISNN), the future of personalized nutrition should include three levels: “(1) conventional nutrition based on general guidelines for population groups by age, gender, and social determinants; (2) individualized nutrition that adds phenotypic information about the current nutritional status of individuals, and (3) genotype-directed nutrition based on rare or common gene variations” (6). This statement highlights future efforts from general dietary guidelines to stratified and genotype-based personalized approaches.

Genotype-based nutritional intervention has been evidently useful for individuals with genetic defects and has helped them effectively improve their health (7), especially for individuals with rare genetic disorders such as phenylketonuria, galactosemia, and vitamin D-resistant rickets (8). Till date, with the aid of candidate-gene approaches or genome-wide association studies (GWAS), several single nucleotide polymorphisms (SNPs) have been identified to have influence over the absorption, distribution, metabolism, excretion, and signal transduction of macronutrients and micronutrients (9–12). In this regard, a few genotypes have been further highlighted to discriminate individuals based on sensitivity to certain nutritional interventions, expanding the understanding of the implementation of personalized nutrition (13–20).

**Figure 1:**
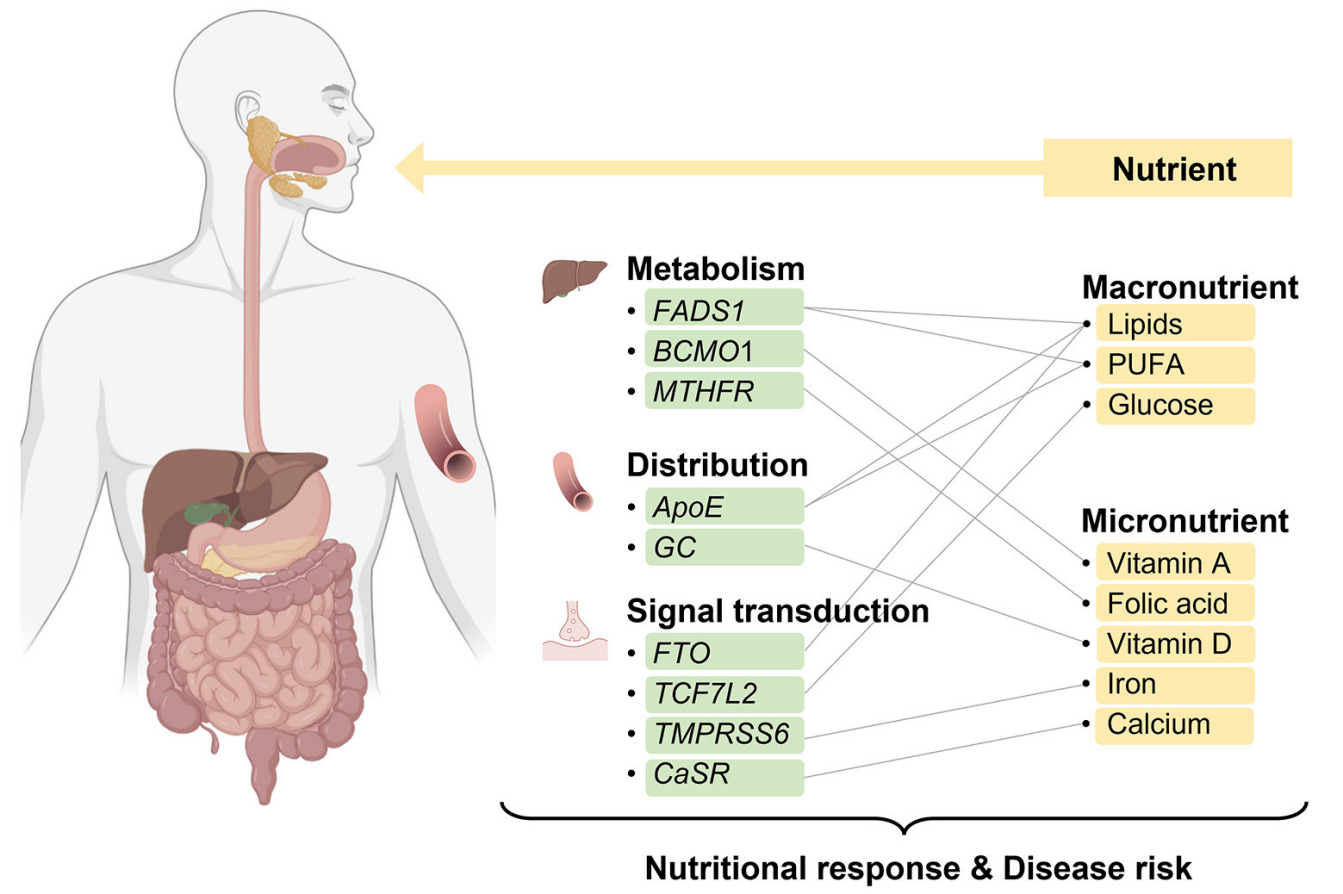
Overview of interactions of nutrients and genes involved in the nutritional metabolism, distribution, and signal transduction. Created with BioRender.com.

In this review, we summarize the critical genotypes that influence the levels of macronutrients and micronutrients and provide the evidence that genotypes may interact with nutritional supplementation or diet to alter nutritional response or disease risk (Figure 1; Table 1). The aim of this review is to provide insights into the use of nutritional genotypes to guide individuals to determine appropriate nutritional supplementation and ultimately achieve optimal health benefits.

**Table 1:** Summary of nutrigenetic evidence for the role of nutritional supplementation in personalized nutrition.

**Nutrition**	**Gene**	**SNP**	**Common Allele**	**Risk Allele**	**Risk Genotype**	**Health issue**	Studies for genotype-based personalized nutrition^*^	**Ref**
Lipid	*ApoE*	rs429358 rs7412	T T	C C	ε4: C/C	Cardiovascular and Alzheimer's Disease	(1) *ApoE* non-ε4 → (Mediterranean diet → ↓AD risk)	(18)
							(2) *ApoE* non-ε4 → (PUFA → ↓cognitive decline)	(19, 20)
							(3) *ApoE* ε4 → (Saturated fat → ↑AD risk)	(21)
							(4) *ApoE* ε4 → (High glycemic load snack → ↑cognitive decline)	(11)
	*FTO*	rs1121980	C	T	CT, TT	Obesity and appetite	(1) *FTO* rs1121980 (T) ↑(Personalized intervention → weight loss)	(25–27)
n-3 PUFA	*FADS1*	rs174546	C	T	CT, TT	Cardiovascular disease	(1) *FADS1* rs174546 (TT) → (ALA → ↓ischemic stroke)	(12)
							(2) *FADS1* rs174546 (TT) → (DHA supplementation → ↑erythrocyte DHA)	(35)
Glucose	*TCF7L2*	rs7903146	C	T	CT, TT	Diabetes and obesity	(1) *TCF7L2* rs7903146 (TT) → (OGTT → ↑plasma free fatty acid, glucose response)	(13)
Vitamin A	*BCOM1*	rs12934922	A	T	AT, TT	Dry eyes, delayed growth, and infectious diseases	(1) *BCOM1* rs12934922 (A/T)↑(dietary carotenoid → plasma β-carotene response)	(47)
							(2) *BCOM1* rs12934922 (T)↑(dietary carotenoid → plasma and prostate carotenoid response)	(14)
Folic acid	*MTHFR*	rs1801133	C	T	CT, TT	Fetal pathologies, neural tube disease, anemia, hypertension	(1) *MTHFR* rs1801133 (TT) ↓ (folic acid supplementation → ↓stroke risk)	(15)
							(2) *MTHFR* rs1801133 (TT) → (Vitamin B2 supplementation → ↓blood pressure)	(58, 59)
							(3) *MTHFR* rs1801133 (TT) → (Vitamin B2 supplementation → ↓ homocysteine)	(60)
Vitamin D	*GC CYP2R1*	rs4588 rs10741657	C A	A G	CA, AC, AG, GG	Rickets, osteoporosis, diabetes mellitus, tuberculosis and chronic obstructive pulmonary disease	(1) *GC* rs4588 (A) + *CYP2R1* rs10741657 (G) ↓(UVB treatment or vitamin D_3_ fortified food → plasma 25(OH)D response)	(65)
							(2) *GC* rs4588 (CC) ↑(VD_3_ supplementation → plasma 25(OH)D response)	(67, 68)
Fe	*TMPRSS6*	rs855791	C	T	CT, TT	Iron-deficiency anemia, diabetes mellitus	(1) *TMPRSS6* rs855791 (T)↓(iron supplementation → serum hemoglobin, iron and ferritin response)	(69)
Ca	*CaSR*	rs17251221	A	G	AG, GG	Nephrolithiasis, and Alzheimer's disease	Unknown	

* For simplicity, studies were expressed as “genotype(s) modify (diet → outcome).” For example, “BCO1-rs12934922 (A/T)↑(dietary carotenoid → plasma carotenoid response)” means that BCO1-rs12934922 (A/T) genotype showed stronger response to dietary carotenoid for plasma β-carotene, compared to its counterpart genotype. AD: Alzheimer's disease; PUFA: polyunsaturated fatty acid; ALA: α-linoleic acid; OGTT: oral glucose tolerancetest.

## Genotype-based supplementation of macronutrients

### Lipids and *ApoE* polymorphism

Lipids are an essential class of hydrophobic biomolecules that include phospholipids, sterols, and triglycerides (TG). They are involved in maintaining energy balance, sustaining vital processes, controlling food intake, and regulating growth and reproduction. However, intake of excessive lipids, such as TG and low-density lipoprotein cholesterol (LDL), may lead to an increased risk of metabolic diseases.

**Figure 2:**
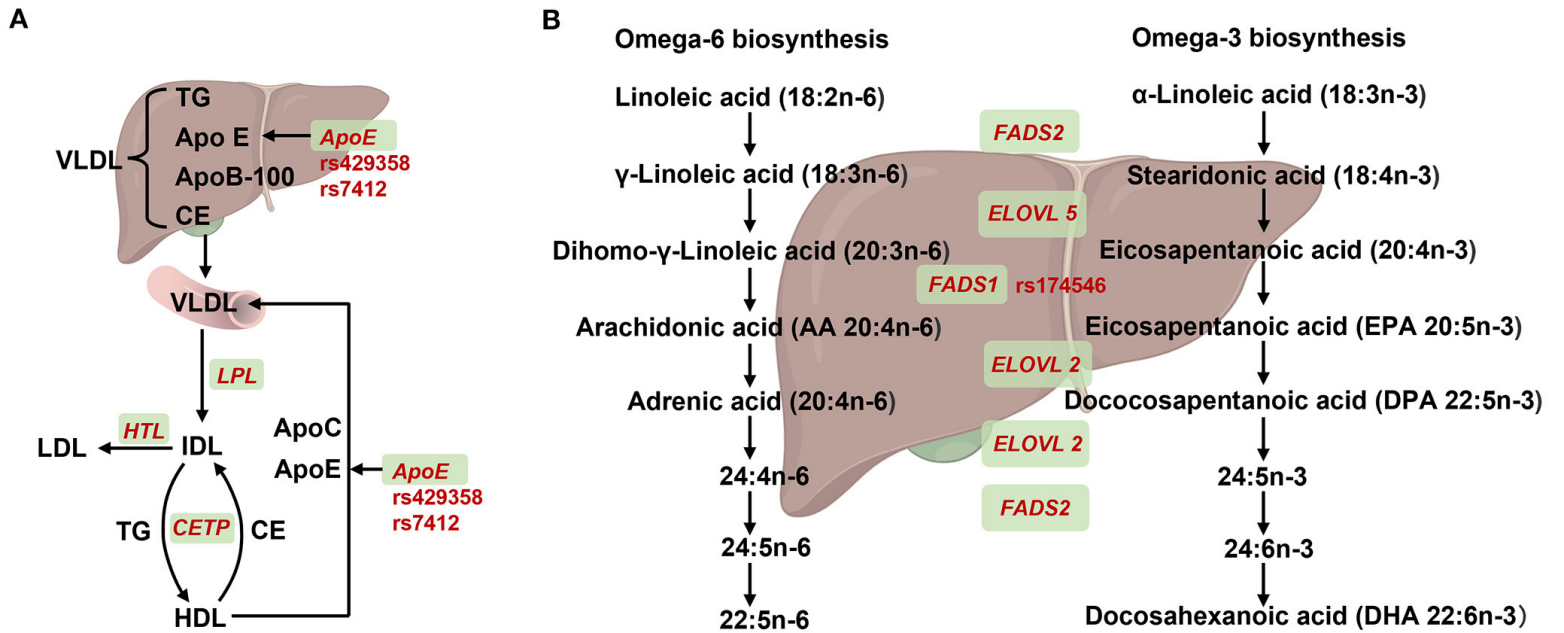
Summary of SNPs for *APOE* and *FADS1* in lipids synthesis or metabolism. **(A)** Effect of *ApoE* recycling and aggregation on lipoprotein. Very low-density lipoprotein (VLDL) containing triglycerides (TG), apolipoprotein E (encoded by *ApoE*), apolipoprotein B-100 (encoded by *ApoB-100*) and cholesteryl ester (CE) that was synthesized in the liver and then secreted into the blood. The VLDL partially degreased by lipoprotein lipase (encoded by *LPL*) and converted to intermediate-density lipoprotein (IDL). On one hand, IDL was hydrolyzed into low-density lipoprotein (LDL) by triglyceride lipase (encoded by *HTL*). On the other hand, IDL and high-density lipoprotein (HDL) could mutual conversion by exchanging TG and CE with cholesteryl ester transfer protein (encoded by *CETP*). In addition, nascent VLDL could be formed by acquisition of ApoE and ApoC from HDL. **(B)** Overview of *FADS1* and *FADS2* genes in desaturation steps necessary for polyunstatured fatty acid (PUFA) biosynthesis. For omega-6 biosynthesis, linoleic acid (LA) was absorbed from diet and converted to γ-Linoleic acid, Dihomo-γ-Linoleic acid by fatty acid synthase 2 (encoded by *FAS2*) and the elongase of very long chain fatty acid 5 (encoded by *ELOVL5*). Subsequently, the dihomo-γ-Linoleic acid was converted into long chain highly unsaturated PUFAs, including arachidonic acid (AA), adrenic acid, 24: (4n-6), 24: 5n-6, 22:5n-6 and 22:5n-6 PUFAS by fatty acid synthase 1 (encoded by *FADS1*), *ELOVL2* and *FADS2*. For Omega-3 biosynthesis, α-Linoleic acid was absorbed from diet and converted to stearidonic acid and eicosapentanoic acid by *FADS2* and *ELOVL5*, respectively. Subsequently, the eicosapentanoic acid was converted into long chain highly unsaturated PUFAs, including eicosapentanoic acid (EPA), dococosapentanoic acid (DPA), 24:5n-3, 24:6n-3 and docosahexanoic acid (DHA) by *FADS1, ELOVL2* and *FADS2*, respectively. Created with BioRender.com.

Apolipoprotein (ApoE), its three isoforms, namely ApoE2, ApoE3, and ApoE4 encoded by ε2, ε3, and ε4 haplotype, respectively, plays key roles in the transport of cholesterol and functioning of cholesterol and other lipids in the brain (Figure 2A). This haplotype system comprises two non-synonymous SNPs rs429358 (T/C) and rs7412 (T/C) in the exon of *ApoE*, where ε2 haplotype is represented by TT, ε3 represented by TC, and ε4 represented by CC. Furthermore, rs769449 exhibits strong linkage disequilibrium with rs429358 (21). The ε4 allele, contributing to impaired LDL binding, has been positively associated with an increased risk of numerous diseases, such as cardiovascular diseases (CVDs) and Alzheimer's disease (AD). A study of 544 patients with hypertension or coronary heart disease indicated that *ApoE* ε4 carriers exhibited high total cholesterol, TG, and LDL levels (22). In addition, the *ApoE* ε4 allele has been reported as the strongest genetic risk factor for sporadic AD in genome-wide association meta-analyses (9).

Evidence suggests that the genotyping for *ApoE* may help develop a highly targeted approach to disease prevention. Adherence to Mediterranean diet, a well-known healthy dietary pattern, may lower AD related anatomical or clinical symptoms in individuals without ε4 genotype (23). In addition, long-chain n-3 polyunsaturated fatty acids (n-3 PUFA) may have a protective role in individuals without ε4 (24, 25). On the contrary, the association of unfavorable diet with increased disease risk in ε4 carriers has been reported. Diets high in saturated fatty acids may increase AD risks by seven folds in ε4 carriers than in non-carriers (26). Moreover, compared to ε4 non-carriers, afternoon snacks with a high glycemic load have been significantly associated with cognitive decline in ε4 carriers (13). These studies suggested that ε4 carriers might require a diet containing healthier lipids to prevent AD onset.

### Lipids and *FTO* polymorphism

Fat mass and obesity-associated gene (*FTO*), involved in the expression of fat deposition and metabolism-related hormones and genes, is the first gene associated with obesity. Furthermore, SNP rs9939609, as well as the proxy SNP rs1121980 of *FTO* has been correlated with obesity and diabetes (27). Maha et al. showed that *FTO* rs9939609 A/A genotype was significantly associated with impaired fasting glucose and insulin resistance (28). In addition, higher serum leptin and lower high-density lipoprotein levels were observed in the homozygotes of the *FTO* rs9939609 risk genotype (AA) compared to those with the TT genotype in overweight adults (29), suggesting the need of precise interventions for these high-risk population. In fact, individuals genetically predisposed to obesity particularly benefit by regulating dietary intake (30, 31) or following personalized diet (32), suggesting a crucial role of *FTO* in personalized nutrition.

### n-3 PUFA and *FADS* polymorphism

n-3 PUFA, such as eicosapentaenoic acid (EPA) and docosahexaenoic acid (DHA) are essential for maintaining health by contributing to organ development, membrane fluidity, and inflammation status. EPA and DHA can be synthesized by desaturases and elongases through the PUFA biosynthetic pathway, wherein fatty acid desaturase (FADS) enzymes including FADS1 and FADS2, are rate-limiting step enzymes (33) (Figure 2B).

GWAS on serum n-3 and n-6 PUFA showed that *FADS1*/*FASD2* strongly affected serum PUFA levels (8). Additionally, Szilvia et al. demonstrated that all haplotypes carrying the *FADS1* rs174546 minor allele were associated with lower Δ-5 desaturases (D5D) activity, which has been associated with plasma long-chain PUFA and lipid levels in 1,144 European adolescents (34). Previous studies also indicated that the *FADS1* rs174546 C/T allele was an important determinant of plasma TG concentrations (35). In addition, several other *FADS1* SNPs have been significantly associated with PUFA levels, such as rs174537 (including three variants G/G, G/T, and T/T) and rs174547 (C/T) (36–38).

Cumulative evidence suggested that the link between PUFA intake and the risk of CVD could be altered using genetic differences in *FADS* (39). In a large cohort study, α-linoleic acid (ALA) intake was inversely associated with ischemic stroke in rs174546 TT genotype carriers with low D5D activity (14). Moreover, the findings that infants who received fish oil supplements exhibited significantly higher erythrocyte DHA levels were only noticed in homozygous for the minor rs174546 as well as other linkage disequilibrium SNPs (40). Nevertheless, more studies are required to employ *FADS1* genotyping to personalized nutrition.

### Glucose and *TCF7L2* polymorphism

Glucose metabolic disorders may play an important role in the pathogenesis of diabetes, CVD, cerebrovascular diseases, and hypertension. SNPs in genes such as *TCF7L2, GCKR, G6PC2*, and *ALOX5* play an important role altering in glucose metabolism (41–45). Among them, *Transcription Factor-7-Like-2* (*TCF7L2*), which belongs to the T-cell factor/lymphoid enhancer factor (TCF/LEF) family, is the most common susceptibility gene for type 2 diabetes mellitus (T2DM) (46). Studies have further identified that *TCF7L2* rs7903146 polymorphism was associated with glucose homeostasis and obesity-related parameters. A meta-analysis of 115,809 subjects indicated that *TCF7L2* rs7903146 polymorphism was significantly associated with susceptibility to T2DM (47). It was also reported that *TCF7L2* rs7903146 T allele was associated with elevated glycated-hemoglobin levels in healthy individuals (11). Moreover, Li et al. indicated significant associations between rs7903146 and body mass index or waist circumference and elevated blood glucose levels (48).

The interaction of *TCF7L2* and diet on glucose homeostasis has been actively investigated. Lu et al. indicated that *TCF7L2* rs7903146 polymorphism affected glucose tolerance and free fatty acid metabolism in adults. They also found that monounsaturated fatty acid concentrations and percentages were greater in females with the TT genotype than in those with the CC genotype in oral glucose tolerance test, and that TT carriers with high HOMA-IR exhibited significantly higher fasting free fatty acid concentrations, lower disposition index, and elevated glucose area under the curve than CC carriers (15). These results indicated that *TCF7L2* SNP-based intervention can be helpful for regulating glucose levels using dietary intervention.

## Genotype-based supplementation of micronutrients

### Vitamin A and *BCMO1* polymorphism

**Figure 3:**
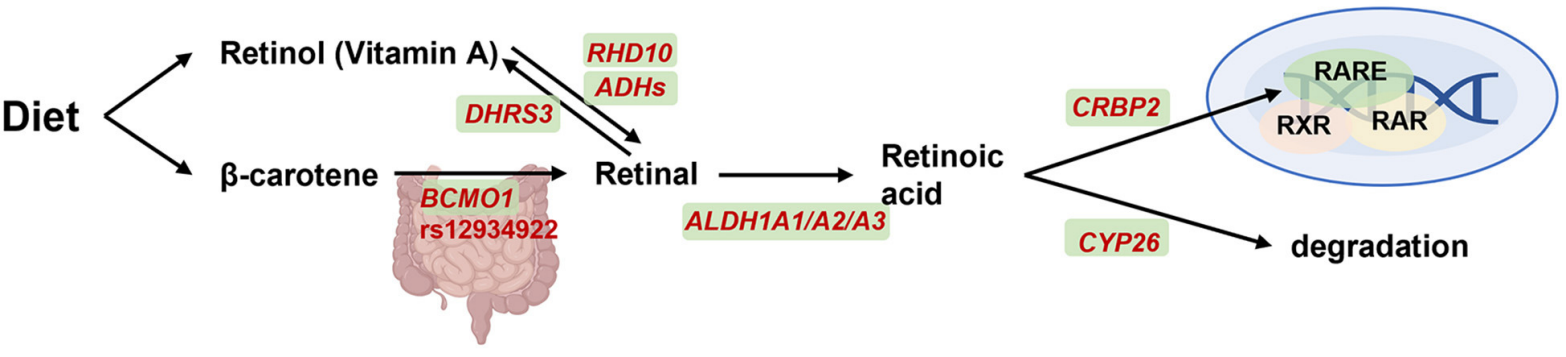
*BCMO1* on transport, signaling and degradation of vitamin A metabolism. Retinol from diet is transported throughout the vasculature and oxidized by retinol dehydrogenase 10 (encoded by *RHD10*) and alcohol dehydrogenases (encoded by *ADHs*) into retinal and then oxidized into retinoic acid (RA) by aldehyde dehydrogenase 1A1, A2, and A3 (encoded by *ALDH1A1-3*). Retinal was also reversible generation to vitamin A by short-chain dehydrogenase/reductase (encoded by *DHRS3*). β-carotene was another alternative pathway which conversed to retinal by β-carotene-15,15-dioxygenase (encoded by *BCMO1*). Subsequently, on one hand, RA will be combined with binding to cellular retinol binding protein 2 (CRBP2) and then activate gene expression together with retinoid-X-receptor (RXR), retinoic acid receptor (RAR) and retinoic acid receptor element (RARE). On another hand, RA can diffuse from cell and degraded by cytochrome P450 26A1 (CYP26) enzyme. Created with BioRender.com.

Vitamin A, a fat-soluble vitamin, is derived from two different sources: the preformed vitamin A from animal-based food, and carotenoids with provitamin A activity from plant-based products (49). The conversion of β-carotene from dietary carotenoids to retinal is the first step in the utilization of vitamin A (Figure 3) (50). Vitamin A plays an important role in maintaining visual function, promoting cell proliferation and differentiation, enhancing immune function, promoting body growth and bone metabolism, and improving hemoglobin levels. Vitamin A deficiency may cause dry eye, infectious diseases such as measles, malaria, diarrhea and respiratory infections, resulting in severe complications such as growth retardation, anemia and even death.

β-carotene 15,15'-monooxygenase 1 (BCMO1) is the most critical enzyme involved in retinoid metabolism (51). Leung et al. identified two common nonsynonymous SNPs (R267S: rs12934922; A379V: rs7501331) in the open reading frame of *BCMO1* with 42 and 24% variant allele frequencies, respectively (52). *In vitro* R267S + A379V double mutant exhibited reduced BCMO1 activity by 57%, whereas female carriers with A379V alone or both R267S and A379V variant alleles exhibited a reduction of 32 and 69%, respectively, in the conversion of beta-carotene to retinol (52). Recently, in a study with 693 Filipino children and adolescents showed that A379V TT variant was inversely related to vitamin A status (53). These results suggested that the polymorphisms in *BCMO1* should be considered for future vitamin A-supplementation recommendations.

Several studies have investigated plasma or tissue response in carriers of vitamin A variants with carotenoid-rich diet. For example, in a 3-week cross-over intervention, 23 healthy subjects were daily provided with juices containing lycopene and β-carotene, and then classified as strong or weak responders based on their plasma carotenoids response profile. *BCMO1* was found to modify these responses, as A/T vs. C genotype in *BCMO1* rs12934922 appeared to be associated with plasma β-carotene changes, while *BCMO2* did not show similar effect partially due to its low frequency (54). Another study examined 11 polymorphisms in putative genes associated with carotenoid metabolism, and *BCMO1* rs12934922 was found to exhibit the strongest effect on carotenoid responses, with the T-allele resulting in elevated lycopene accumulation in plasma and prostate tissue (16). These studies suggested that genetic variations, particularly in *BCMO1* rs12934922, could influence the degree of plasma response to dietary carotenoids, thereby highlighting the need to examine this genotype when considering personalized vitamin A supplementation.

### Folic acid and *MTHFR* polymorphism

Vitamin 9, naturally occurring as folic acid, is synthesized by plants and microorganisms. It cannot be synthesized by humans due to the lack of a complete folate biosynthetic pathway. Furthermore, as folic acid is involved in the biosynthesis of nucleotides, amino acids, and certain vitamins, humans must ingest and absorb folic acid derived from diet (55). Folate deficiency can cause many diseases in both young and old age (56), such as fetal pathologies, neural tube disease (57), anemia (58), and depression (59).

**Figure 4:**
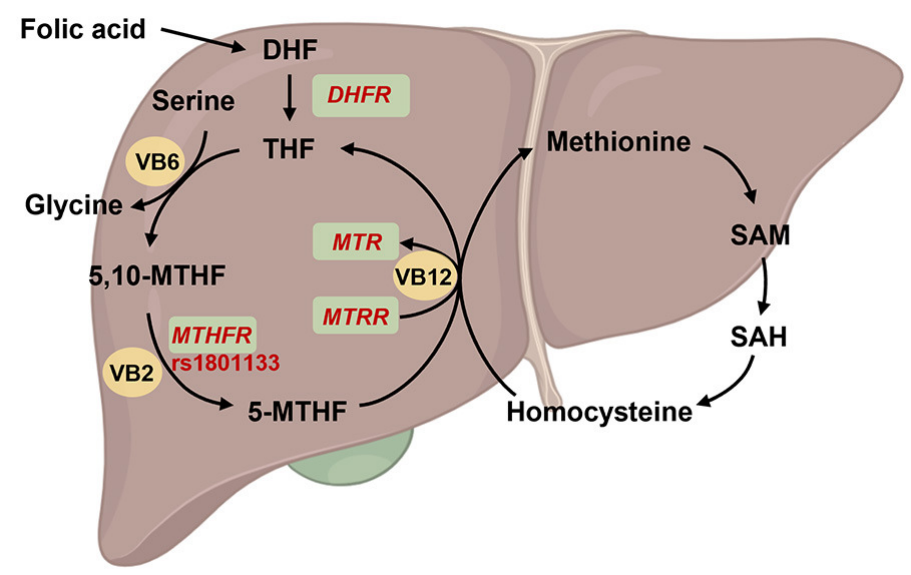
Overview of the function of *MTHFR* on folate metabolism. The folic acid is reverted to dihydrofolate (DHF) and subsequently to tetrahydrofolate (THF) by dihydrofolate reductase (encoded by *DHFR*), and then THF was conversed to methylenetetrahydrofolate (5,10-MTHF). On the other hand, THF involved in the cycle of glycine synthesis from serine with vitamin B6 (VB6) catalysis. Subsequently, 5,10-MTHF was reverted to methylated tetrahydrofolate (5-MTHF) by methylenetetrahydrofolate reductase (encoded by *MTHFR*) and vitamin B2 (VB2) catalysis. 5-MTHF will be transform into THF and participated into homocysteine-methionine cycle by 5-methyltetrahydrofolate-homocysteine methyltransferase (encoded by *MTR*) and methionine synthase reductase (encoded by *MTRR*) under vitamin B12 (VB12) catalysis. Created with BioRender.com.

As 5,10-methylenetetrahydrofolate reductase (MTHFR) is a key enzyme in folic acid metabolism, its polymorphism can decrease enzyme activity by 60%, resulting in disorders associated with folate metabolism as well as a variety of other diseases (Figure 4). Till date, 14 rare mutations with severe enzymatic deficiencies and one mutation rs1801133 (C677T) with a milder enzymatic deficiency in *MTHFR* have been reported (60, 61). In addition, serum folic acid concentrations were lower in individuals with the *MTHFR* rs1801133 TT genotype than in individuals with the CC or CT genotypes (62). Moreover, the risk genotype of rs1801133 has been found to be associated with various diseases, including diabetes, CVDs, cancer, and vascular disorders (62, 63). Several studies have also revealed that *MTHFR* polymorphisms might be associated with the elevation levels of homocysteine, thus exacerbating CVD risk (64).

Studies on *MTHFR* have been successfully used to develop disease prevention strategies. In the China Stroke Primary Prevention Trial, the antihypertensive drug enalapril, in addition to folic acid supplementation, significantly reduced the risk of stroke in adults with hypertension, while *MTHFR* rs1801133 TT genotype did not benefit those with low baseline folic acid levels even upon folic acid supplementation, implying that higher dosage of folic acid is needed for individuals at risk of CVD with disadvantageous *MTHFR* genotype (17). Interestingly, riboflavin (vitamin B2), the co-factor for MTHFR in folate metabolism, has also been used to regulate blood pressure (65, 66) or homocysteine levels (67), but such beneficial effects were only reported in the rs1801133 TT group, suggesting that vitamin B2 might compensate for the loss of MTHFR activity. In summary, these studies help improve the implementation of more precise and effective folic acid recommendations while considering *MTHFR* polymorphisms.

### Vitamin D and *GC* polymorphism

**Figure 5:**
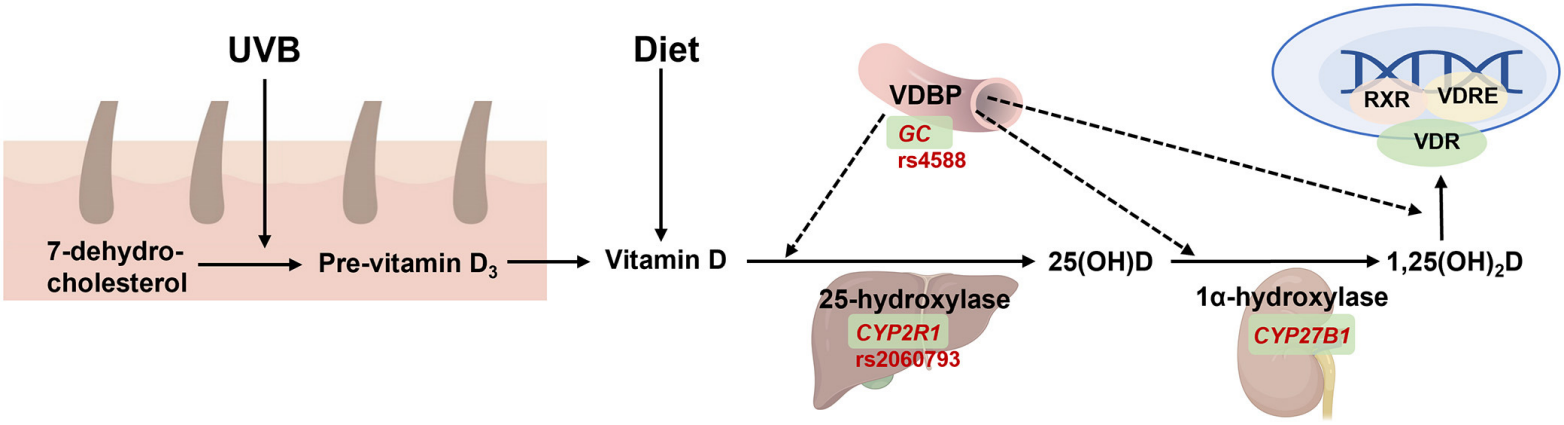
The function of *GC* on vitamin D synthesis, transport, and metabolism. 7-dehydrocholestrol is converted to pre-vitamin D3 and then to vitamin D in the skin under ultraviolet radiation b (UVB) exposure. Vitamin D is also absorbed from diet and then transferred by binding with vitamin D binding protein (VDBP, encoded by *GC*) into liver to form 25(OH)D by 25-hydroxylase (encoded by *CYP2R1*), and then transferred into the kidney by VDBP which form the active metabolite 1,25(OH)2D by 1α-hydroxylase (encoded by *CYP27B1*). The latter is then transferred to targeted tissue to combine with binding to vitamin D receptor (VDR), and then activate gene expression together with retinoid-X-receptor (RXR) and vitamin D response element (VDRE). Created with BioRender.com.

Vitamin D is an important fat-soluble vitamin, which is primarily synthesized in the skin upon exposure to ultraviolet B radiation (UVB) in sun exposure. In the circulation, vitamin D and its products are transferred into the liver and kidneys by vitamin D binding proteins (VDBP, encoded by *GC*) (Figure 5). Vitamin D regulates the absorption of calcium and phosphorus, maintains the levels of blood calcium and phosphorus levels, and promotes normal bones and teeth development. However, the widespread distribution of VDBPs in the body indicates its unconventional role beyond calcium and phosphorus regulation, including regulation of cell proliferation and differentiation, synthesis, and secretion of cytokines and other hormones. Moreover, vitamin D is critical in preventing cancer, immune diseases, and diseases of the endocrine system.

Several VDBP polymorphisms have been identified to affect vitamin D levels that contribute to health outcomes (68), with the most common polymorphisms being rs4588 and rs7041 in *GC*, which may correlate with serum vitamin D levels (70, 71). Spyridon et al. demonstrated that mothers with the CC genotype for rs2298850 and rs4588 polymorphisms showed elevated 25(OH)D concentrations (72). Dong et al., also showed that *GC* SNPs including rs17467825, rs4588, rs2282679, rs2298850, and rs1155563, were significantly associated with maternal 25(OH)D concentrations (73). Furthermore, a recent study confirmed the rs4588 and rs7041 polymorphisms were more frequent in the T2DM patients, compared to the control group (74). These results showed that rs4588, rs7041, and other *GC* SNPs may be correlated with not only serum vitamin D status but also the risk of T2DM.

Ongoing studies have shown that genetic predisposition related to the vitamin D metabolic pathway could modify the response of 25(OH)D after vitamin D supplementation or UVB exposure. Nimitphong et al. (69) reported that *GC* rs4588 CC carriers exhibited an increase in 25(OH)D levels compared to those with risk genotypes CA or AA, upon daily vitamin D_3_ supplementation for 3 months; however, this interaction was not observed for vitamin D_2_ supplementation. Nonetheless, when more genes were included, the effects of genetic variants persisted. In addition, Nissen et al. indicated that carriers of all four risk alleles of *GC* rs4588 (A) and *CYP2R1* rs2060793 (G) were the least benefitted from the consumption of vitamin D_3_-fortified food or UVB exposure (73). Moreover, in a larger randomized controlled trial, Yao et al. verified that the subjects with the risk genotype of *GC* exhibited a significantly lower increase in 25(OH)D response after 20-week vitamin D_3_ supplementation, and that the genetic factors, evaluated as genetic risk score encompassing four variants of *GC, VDR* and *CYP2R1* genes exerted greater impact on 25(OH)D response than did non-genetic factors (75). Therefore, these studies suggested that genetic factors, perhaps more important than non-genetic factors, could provide valuable insights for appropriate vitamin D recommendations, as those with the risk genotype of *GC* and other vitamin D metabolism-related genes may require increased vitamin D consumption or UVB exposure.

### Iron and *TMPRSS6* polymorphism

Iron, as an essential trace element, regulates vital physiological processes in the body, including DNA synthesis, electron transport and oxygen transport. As iron homeostasis and metabolism are tightly regulated, iron surplus or deficiency can lead to various diseases (76).

Transmembrane protease serine 6 (TMPRSS6), which is responsible for iron absorption and recycling, is critical for iron homeostasis. GWAS have shown that *TMPRSS6* variants were most associated with serum iron, soluble transferrin receptor, and hemoglobin levels (12). Specifically, the *TMPRSS6* rs855791 T genotype with a non-synonymous amino acid substitution (A736V) reduced the enzymatic activity of TMPRSS6. Furthermore, each copy of the minor alleles A and G of the rs855791 polymorphism decreased serum ferritin by 4.50 g/L and by 5.00 μg/L, respectively, in under-2-year-old children in Indonesia (77). Pei et al. also demonstrated that homozygotes with the *TMPRSS6* rs855791 C genotype mitigated iron deficiency anemia in women at reproductive age, especially in those with menorrhagia (78). A recent study showed that the rs855791polymorphism was significantly associated with decreased iron levels in the participants from Saudi Arabia (79). Moreover, Gan et al. revealed that the rs855791 and rs4820268 polymorphisms were both significantly associated with plasma ferritin, hemoglobin levels, iron overload risk, and T2DM risk in a Chinese population (80), suggesting the role of *TMPRSS6* in iron homeostasis as well as disease risk.

Since not only iron deficiency, but also iron overload can exert adverse effects, it is critical to tailor personalized iron supplementation to ensure iron homeostasis. De Falco et.al. (81) indicated that for patients with persistent iron-deficiency anemia, carriers of the *TMPRSS6* rs855791 risk allele (T) exhibited reduced serum iron changes upon oral iron supplementation. Nevertheless, further studies are warranted to investigate the effects of *TMPRSS6* in the management of iron nutritional status.

### Calcium and *CaSR* polymorphism

Calcium is one of the primary constituents of human body, and its active form is vital to maintain the integrity of cell membranes, regulate excitation of muscles, and monitor various functions of cells. Calcium deficiency is associated with diseases of a variety of tissues and systems, such as bone, endocrine, cardiovascular and cerebrovascular, nervous, digestive, urinary, reproductive, and nervous systems (82), all of which could be prevented with the use of calcium supplementation (83, 84).

Calcium-sensing receptors (CaSRs), members of the family of G protein-coupled receptors, are mainly located in the parathyroid gland and maintain the homeostasis of calcium by regulating the secretion of parathyroid hormone (85). A GWAS of 20,611 individuals of European ancestry showed that the rs17251221 polymorphism was associated with elevated serum calcium levels and accounted for 0.54% of the variance, and that the G allele of the same polymorphism was also associated with higher serum magnesium levels, lower serum phosphate levels, and lower bone mineral density in the lumbar spine (86). Another genome-wide meta-analysis on serum calcium revealed that a missense variant rs1801725 accounted for 1.26% of the variance in serum calcium levels, with the strongest association exhibited in individuals of European descent, whereas similar association was observed for rs17251221 in individuals of Indian Asian descent (87). However, to the best our knowledge, no studies have determined the effects of the interaction between the *CaSR* genotype and dietary intervention on calcium levels or health outcomes, thereby necessitating further investigation for the precise management of calcium levels.

## Conclusion and prospect

With the rapidly progressing fields of nutrigenetics, accumulation of genetic variants has been identified to influence both macronutrient and micronutrient levels, as well as individual responses to dietary intake. Such variants are valuable to develop appropriate personalized dietary interventions, thus ensuring the transition of generic dietary guidelines to genotype-directed nutrition.

However, several challenges may hinder the widespread adoption of personalized nutrition. Firstly, nutrition-related diseases, such as CVDs and T2DM, are considered as polygenic in nature, and are thus influenced by multiple genes with small or medium effects. Therefore, polygenic risk scores of more than a few variants may provide more information to predict the outcome of personalized nutritional intervention (88). Secondly, diseases result from the interaction of genes and environmental factors. For example, individuals with genetic predisposition to obesity may have increased odds of weight gain compared to general population when they consume the same amount of sugar-sweetened beverages (89). In this regard, deep phenotyping using advanced “omics” technologies including epigenomics, transcriptomics, proteomics, metabolomics, and microbiome may help to discover the underlying gene-environment interactions and explain the missing hereditary (90, 91). Thirdly, “omics” technologies generate a large amount of data, requiring the use of advanced analytical methods such as machine learning (92, 93), which has been applied in multiple stages of personalized nutrition, including blood glucose monitoring (94), body weight management (95), disease risk assessment (96) and nutritional management (97). For example, by integrating blood biomarkers, diet, anthropometrics, and gut microbiota, a machine-learning algorithm could accurately predict the postprandial glycemic response, and therefore assisting in blood glucose homeostasis (4). Fourthly, the efficient implementation of personalized nutrition requires accurate intervention of health professionals and good compliance from individuals, thus requiring novel digital tools or tracking devices to bridge the gap between them (98).

In conclusion, genotype-based nutritional studies have highlighted the critical role of SNPs in the regulation of macronutrient and micronutrient levels, which are fundamental for health. Although further studies are required for the adequate implementation of personalized nutrition into healthcare research and practice, current evidence indicates the necessity of incorporating more genetic variants into personalized nutritional interventions to achieve improved nutrient levels and health outcomes and reduce the burdens of nutritional disorders on healthcare services.

## Author contributions

JK, YY, YL, and JD designed the manuscript. FeiW, JZ, HZ, ML, BD, RL, FenW, DH, and GL performed the literature review. FeiW and JZ wrote the manuscript. JC, RZ, and XD revised the manuscript. All authors contributed to the article and approved the submitted version.

## Conflict of interest

Authors HZ and YL are employed by Sequanta Technologies Co., Ltd. The remaining authors declare that the research was conducted in the absence of any commercial or financial relationships that could be construed as a potential conflict of interest.

## Publisher's note

All claims expressed in this article are solely those of the authors and do not necessarily represent those of their affiliated organizations, or those of the publisher, the editors and the reviewers. Any product that may be evaluated in this article, or claim that may be made by its manufacturer, is not guaranteed or endorsed by the publisher.

## References

[1] Elia M. Defining, recognizing, and reporting malnutrition. Int J Low Extrem Wounds. (2017) 16:230–7. 10.1177/1534734617733902 29145755

[2] Cederholm T Barazzoni R Austin P Ballmer P Biolo G Bischoff SC. Espen guidelines on definitions and terminology of clinical nutrition. Clin Nutr. (2017) 36:49–64. 10.1016/j.clnu.2016.09.004 27642056

[3] Yardley JE Campbell MD. Moving toward precision medicine with diabetes, exercise and physical activity. Can J Diabetes. (2020) 44:679. 10.1016/j.jcjd.2020.10.008 33279095

[4] Zeevi D Korem T Zmora N Israeli D Rothschild D Weinberger A. Personalized nutrition by prediction of glycemic responses. Cell. (2015) 163:1079–94. 10.1016/j.cell.2015.11.001 26590418

[5] Kan J Ni J Xue K Wang F Zheng J Cheng J. Personalized nutrition intervention improves health status in overweight/obese chinese adults: a randomized controlled trial. Front Nutr. (2022) 9:919882. 10.3389/fnut.2022.919882 35811975 PMC9258630

[6] Ferguson LR De Caterina R Gorman U Allayee H Kohlmeier M Prasad C. Guide and position of the international society of nutrigenetics/nutrigenomics on personalised nutrition: part 1 - fields of precision nutrition. J Nutrigenet Nutrigenomics. (2016) 9:12–27. 10.1159/000445350 27169401

[7] Marcum JA. Nutrigenetics/nutrigenomics, personalized nutrition, and precision healthcare. Curr Nutr Rep. (2020) 9:338–45. 10.1007/s13668-020-00327-z 32578026

[8] de Toro-Martin J Arsenault BJ Despres JP Vohl MC. Precision nutrition: a review of personalized nutritional approaches for the prevention and management of metabolic syndrome. Nutrients. (2017) 9:913. 10.3390/nu9080913 28829397 PMC5579706

[9] Serrano-Pozo A Das S Hyman BT. Apoe and Alzheimer's disease: advances in genetics, pathophysiology, and therapeutic approaches. Lancet Neurol. (2021) 20:68–80. 10.1016/S1474-4422(20)30412-9 33340485 PMC8096522

[10] Coltell O Sorli JV Asensio EM Barragan R Gonzalez JI Gimenez-Alba IM. Genome-wide association study for serum Omega-3 and Omega-6 polyunsaturated fatty acids: exploratory analysis of the sex-specific effects and dietary modulation in mediterranean subjects with metabolic syndrome. Nutrients. (2020) 12:310. 10.3390/nu12020310 31991592 PMC7071282

[11] Podboi ICR Stephenson S Pilic L Graham CA King A Mavrommatis Y. Dietary intake and Tcf7l2 Rs7903146 T allele are associated with elevated blood glucose levels in healthy individuals. Lifestyle Genom. (2021) 14:117–23. 10.1159/000518523 34515148

[12] Tanaka T Roy CN Yao W Matteini A Semba RD Arking D. A Genome-wide association analysis of serum iron concentrations. Blood. (2010) 115:94–6. 10.1182/blood-2009-07-232496 19880490 PMC2803694

[13] Gentreau M Raymond M Chuy V Samieri C Artero S. High Glycemic load is associated with cognitive decline in apolipoprotein E E4 allele carriers. Nutrients. (2020) 12:3619. 10.3390/nu12123619 33255701 PMC7761247

[14] Hellstrand S Ericson U Gullberg B Hedblad B Orho-Melander M Sonestedt E. Genetic variation in fads1 has little effect on the association between dietary pufa intake and cardiovascular disease. J Nutr. (2014) 144:1356–63. 10.3945/jn.114.192708 25008580 PMC4130826

[15] Lu J Varghese RT Zhou L Vella A Jensen MD. Glucose tolerance and free fatty acid metabolism in adults with variations in Tcf7l2 Rs7903146. Metabolism. (2017) 68:55–63. 10.1016/j.metabol.2016.11.018 28183453 PMC5308561

[16] Moran NE Thomas-Ahner JM Fleming JL McElroy JP Mehl R Grainger EM. Single nucleotide polymorphisms in beta-carotene oxygenase 1 are associated with plasma lycopene responses to a tomato-soy juice intervention in men with prostate cancer. J Nutr. (2019) 149:381–97. 10.1093/jn/nxy304 30801647 PMC6398392

[17] Huo Y Li J Qin X Huang Y Wang X Gottesman RF. Efficacy of folic acid therapy in primary prevention of stroke among adults with hypertension in China: the csppt randomized clinical trial. JAMA. (2015) 313:1325–35. 10.1001/jama.2015.2274 25771069

[18] Grimaldi KA van Ommen B Ordovas JM Parnell LD Mathers JC Bendik I. Proposed guidelines to evaluate scientific validity and evidence for genotype-based dietary advice. Genes Nutr. (2017) 12:35. 10.1186/s12263-017-0584-0 29270237 PMC5732517

[19] Keathley J Garneau V Zavala-Mora D Heister RR Gauthier E Morin-Bernier J. A systematic review and recommendations around frameworks for evaluating scientific validity in nutritional genomics. Front Nutr. (2021) 8:789215. 10.3389/fnut.2021.789215 35004815 PMC8728558

[20] Parnell LD Blokker BA Dashti HS Nesbeth PD Cooper BE Ma Y. CardioGxE, A catalog of gene-environment interactions for cardiometabolic traits. BioData Min. (2014) 7:21. 10.1186/1756-0381-7-21 25368670 PMC4217104

[21] Villeneuve S Brisson D Marchant NL Gaudet D. The potential applications of apolipoprotein e in personalized medicine. Front Aging Neurosci. (2014) 6:154. 10.3389/fnagi.2014.00154 25071563 PMC4085650

[22] Wang C Yan W Wang H Zhu J Chen H. Apoe polymorphism is associated with blood lipid and serum uric acid metabolism in hypertension or coronary heart disease in a Chinese population. Pharmacogenomics. (2019) 20:1021–31. 10.2217/pgs-2019-0048 31559922

[23] Martinez-Lapiscina EH Galbete C Corella D Toledo E Buil-Cosiales P Salas-Salvado J. Genotype patterns at Clu, Cr1, picalm and apoe, cognition and mediterranean diet: the predimed-navarra trial. Genes Nutr. (2014) 9:393. 10.1007/s12263-014-0393-7 24643340 PMC4026432

[24] Barberger-Gateau P Samieri C Feart C Plourde M. Dietary Omega 3 polyunsaturated fatty acids and Alzheimer's disease: interaction with apolipoprotein E genotype. Curr Alzheimer Res. (2011) 8:479–91. 10.2174/156720511796391926 21605054 PMC3518784

[25] Stonehouse CC Podd A Hill SR. Dha supplementation improved both memory and reaction time in healthy young adults: a randomized controlled trial. Am J Clin Nutr. (2013) 97:1134–43. 10.3945/ajcn.112.053371 23515006

[26] Kivipelto M Rovio S Ngandu T Kareholt I Eskelinen M Winblad B. Apolipoprotein E Epsilon4 magnifies lifestyle risks for dementia: a population-based study. J Cell Mol Med. (2008) 12(6B):2762–71. 10.1111/j.1582-4934.2008.00296.x 18318693 PMC3828889

[27] Peng S Zhu Y Xu F Ren X Li X Lai M. Fto gene polymorphisms and obesity risk: a meta-analysis. BMC Med. (2011) 9:71. 10.1186/1741-7015-9-71 21651756 PMC3118373

[28] Saber-Ayad M Manzoor S El Serafi A Mahmoud I Hammoudeh S Rani A. The Fto Rs9939609 “a” allele is associated with impaired fasting glucose and insulin resistance in Emirati population. Gene. (2019) 681:93–8. 10.1016/j.gene.2018.09.053 30273662

[29] Mehrdad M Doaei S Gholamalizadeh M Fardaei M Fararouei M Eftekhari MH. Association of Fto Rs9939609 polymorphism with serum leptin, insulin, adiponectin, and lipid profile in overweight adults. Adipocyte. (2020) 9:51–6. 10.1080/21623945.2020.1722550 31996075 PMC6999843

[30] Lappalainen T Lindstrom J Paananen J Eriksson JG Karhunen L Tuomilehto J. Association of the fat mass and obesity-associated (Fto) gene variant (Rs9939609) with dietary intake in the finnish diabetes prevention study. Br J Nutr. (2012) 108:1859–65. 10.1017/S0007114511007410 22265018

[31] Huang T Qi Q Li Y Hu FB Bray GA Sacks FM. Fto Genotype, dietary protein, and change in appetite: the preventing overweight using novel dietary strategies trial. Am J Clin Nutr. (2014) 99:1126–30. 10.3945/ajcn.113.082164 24622803 PMC3985215

[32] Carlos CM Marsaux Cyril FM Livingstone KM Santiago NC Rodrigo SC Rosalind F. Can genetic-based advice help you lose weight? Findings from the food4me european randomized controlled. Trial Am J Clin Nutr. (2017) 105:1204–13. 10.3945/ajcn.116.145680 28381478

[33] Santana JDM Pereira M Carvalho GQ Gouveia Peluzio MDC Drumond Louro I Santos DBD. Fads1 and Fads2 gene polymorphisms modulate the relationship of Omega-3 and Omega-6 fatty acid plasma concentrations in gestational weight gain: a Nisami cohort study. Nutrients. (2022) 14:1056. 10.3390/nu14051056 35268031 PMC8912382

[34] Bokor S Dumont J Spinneker A Gonzalez-Gross M Nova E Widhalm K. Single nucleotide polymorphisms in the fads gene cluster are associated with delta-5 and delta-6 desaturase activities estimated by serum fatty acid ratios. J Lipid Res. (2010) 51:2325–33. 10.1194/jlr.M006205 20427696 PMC2903808

[35] AlSaleh A Maniou Z Lewis FJ Hall WL Sanders TA O'Dell SD. Genetic predisposition scores for dyslipidaemia influence plasma lipid concentrations at baseline, but not the changes after controlled intake of N-3 polyunsaturated fatty acids. Genes Nutr. (2014) 9:412. 10.1007/s12263-014-0412-8 24890013 PMC4169063

[36] Mathias RA Sergeant S Ruczinski I Torgerson DG Hugenschmidt CE Kubala M. The impact of fads genetic variants on omega6 polyunsaturated fatty acid metabolism in African Americans. BMC Genet. (2011) 12:50. 10.1186/1471-2156-12-50 21599946 PMC3118962

[37] Metelcova T Vankova M Zamrazilova H Hovhannisyan M Stankova B Tvrzicka E. Fads1 gene polymorphism(S) and fatty acid composition of serum lipids in adolescents. Lipids. (2021) 56:499–508. 10.1002/lipd.12317 34189740

[38] Wang Y Tang Y Ji Y Xu W Ullah N Yu H. Association between Fads1 Rs174547 and levels of long-chain pufa: a meta-analysis. Br J Nutr. (2021) 126:1121–9. 10.1017/S0007114520005103 33331250

[39] Koletzko B Reischl E Tanjung C Gonzalez-Casanova I Ramakrishnan U Meldrum S. Fads1 and Fads2 polymorphisms modulate fatty acid metabolism and dietary impact on health. Annu Rev Nutr. (2019) 39:21–44. 10.1146/annurev-nutr-082018-124250 31433740

[40] Meldrum SJ Li Y Zhang G Heaton AEM D'Vaz N Manz J. Can polymorphisms in the fatty acid desaturase (Fads) gene cluster alter the effects of fish oil supplementation on plasma and erythrocyte fatty acid profiles? An exploratory study. Eur J Nutr. (2018) 57:2583–94. 10.1007/s00394-017-1529-5 28929400

[41] Zhou W Li Y Zhang L Shi Y Wang C Zhang D. Gene-gene interactions lead to higher risk for development of type 2 diabetes in a Chinese Han population: a prospective nested case-control study. Lipids Health Dis. (2018) 17:179. 10.1186/s12944-018-0813-6 30055620 PMC6064617

[42] Hu C Zhang R Wang C Ma X Wang C Fang Q. A genetic variant of G6pc2 is associated with type 2 diabetes and fasting plasma glucose level in the Chinese population. Diabetologia. (2009) 52:451–6. 10.1007/s00125-008-1241-3 19082990

[43] Li X Su J Chen S Lin S Zheng X Wang B. The association between the Rs4987105 of 5-Lipoxygenase (Alox5) gene and gestational glucose metabolism in Chinese population. BMC Res Notes. (2020) 13:102. 10.1186/s13104-020-04953-2 32093765 PMC7041080

[44] Tam CH Ho JS Wang Y Lee HM Lam VK Germer S. Common polymorphisms in Mtnr1b, G6pc2 and Gck are associated with increased fasting plasma glucose and impaired beta-cell function in Chinese subjects. PLoS ONE. (2010) 5:e11428. 10.1371/journal.pone.0011428 20628598 PMC2900202

[45] Hu C Zhang R Wang C Yu W Lu J Ma X. Effects of Gck, Gckr, G6pc2 and Mtnr1b variants on glucose metabolism and insulin secretion. PLoS ONE. (2010) 5:e11761. 10.1371/journal.pone.0011761 20668700 PMC2909258

[46] Del Bosque-Plata L Martinez-Martinez E Espinoza-Camacho MA Gragnoli C. The Role of Tcf7l2 in Type 2 diabetes. Diabetes. (2021) 70:1220–8. 10.2337/db20-0573 34016596 PMC8275893

[47] Lou L Wang J Wang J. Genetic Associations between Transcription Factor 7 Like 2 Rs7903146 polymorphism and type 2 diabetes mellitus: a meta-analysis of 115,809 subjects. Diabetol Metab Syndr. (2019) 11:56. 10.1186/s13098-019-0451-9 31312259 PMC6612193

[48] Li L Wang J Ping Z Li Y Wang C Shi Y. Interaction analysis of gene variants of Tcf7l2 and body mass index and waist circumference on type 2 diabetes. Clin Nutr. (2020) 39:192–7. 10.1016/j.clnu.2019.01.014 30718095

[49] Cheng J Balbuena E Miller B Eroglu A. The role of β-carotene in colonic inflammation and intestinal barrier integrity. Front Nutr. (2021) 8:723480. 10.3389/fnut.2021.723480 34646849 PMC8502815

[50] Lietz G Oxley A Boesch-Saadatmandi C Kobayashi D. Importance of B,B-Carotene 15,15'-Monooxygenase 1 (Bcmo1) and B,B-Carotene 9',10'-Dioxygenase 2 (Bcdo2) in nutrition and health. Mol Nutr Food Res. (2012) 56:241–50. 10.1002/mnfr.201100387 22147584

[51] Li CC Liu C Fu M Hu KQ Aizawa K Takahashi S et al. Tomato powder inhibits hepatic steatosis and inflammation potentially through restoring SIRT1 activity and adiponectin function independent of carotenoid cleavage enzymes in mice. Mol Nutr Food Res. (2018) 62:e1700738. 10.1002/mnfr.201700738 29266812

[52] Leung WC Hessel S Meplan C Flint J Oberhauser V Tourniaire F. Two common single nucleotide polymorphisms in the gene encoding Beta-Carotene 15,15'-monoxygenase alter beta-carotene metabolism in female volunteers. FASEB J. (2009) 23:1041–53. 10.1096/fj.08-121962 19103647

[53] Zumaraga MPP Arquiza J Concepcion MA Perlas L Alcudia-Catalma MN Rodriguez M. Genotype effects on beta-carotene conversion to vitamin a: implications on reducing vitamin a deficiency in the Philippines. Food Nutr Bull. (2022) 43:25–34. 10.1177/03795721211060229 34903070

[54] Wang TT Edwards AJ Clevidence BA. Strong and weak plasma response to dietary carotenoids identified by cluster analysis and linked to beta-carotene 15,15'-monooxygenase 1 single nucleotide polymorphisms. J Nutr Biochem. (2013) 24:1538–46. 10.1016/j.jnutbio.2013.01.001 23517913

[55] Obeid R Oexle K Rissmann A Pietrzik K Koletzko B. Folate status and health: challenges and opportunities. J Perinat Med. (2016) 44:261–8. 10.1515/jpm-2014-0346 25825915

[56] Shulpekova Y Nechaev V Kardasheva S Sedova A Kurbatova A Bueverova E. The concept of folic acid in health and disease. Molecules. (2021) 26:3731. 10.3390/molecules26123731 34207319 PMC8235569

[57] Castano E Pinunuri R Hirsch S Ronco AM. Folate and pregnancy, current concepts: it is required folic acid supplementation?. Rev Chil Pediatr. (2017) 88:199–206. 10.4067/S0370-41062017000200001 28542653

[58] Achebe MM Gafter-Gvili A. How I treat anemia in pregnancy: iron, cobalamin, and folate. Blood. (2016) 129:940–9. 10.1182/blood-2016-08-672246 28034892

[59] Khan R Waqas A Bilal A Mustehsan ZH Omar J Rahman A. Association of maternal depression with diet: a systematic review. Asian J Psychiatr. (2020) 52:102098. 10.1016/j.ajp.2020.102098 32403029

[60] Goyette P Christensen B Rosenblatt DS Rozen R. Severe and mild mutations in cis for the methylenetetrahydrofolate reductase (Mthfr) gene, and description of five novel mutations in Mthfr. Am J Hum Genet. (1996) 59:1268–75. 10.1016/S0921-8777(96)00037-7 8940272 PMC1914869

[61] Goyette P Frosst P Rosenblatt DS Rozen R. Seven novel mutations in the methylenetetrahydrofolate reductase gene and genotype/phenotype correlations in severe methylenetetrahydrofolate reductase deficiency. Am J Hum Genet. (1995) 56:1052–9 7726158 PMC1801446

[62] Li WX Cheng F Zhang AJ Dai SX Li GH Lv WW. Folate deficiency and gene polymorphisms of mthfr, mtr and mtrr elevate the hyperhomocysteinemia risk. Clin Lab. (2017) 63:523–33. 10.7754/Clin.Lab.2016.160917 28271696

[63] Liew SC Gupta ED. Methylenetetrahydrofolate reductase (Mthfr) C677t polymorphism: epidemiology, metabolism, and the associated diseases. Eur J Med Genet. (2015) 58:1–10. 10.1016/j.ejmg.2014.10.004 25449138

[64] Raghubeer S Matsha TE. Methylenetetrahydrofolate (Mthfr), the one-carbon cycle, and cardiovascular risks. Nutrients. (2021) 13:4562. 10.3390/nu13124562 34960114 PMC8703276

[65] Horigan G McNulty H Ward M Strain JJ Purvis J Scott JM. Riboflavin lowers blood pressure in cardiovascular disease patients homozygous for the 677c–>T polymorphism in Mthfr. J Hypertens. (2010) 28:478–86. 10.1097/HJH.0b013e328334c126 19952781

[66] Wilson CP McNulty H Ward M Strain JJ Trouton TG Hoeft BA. Blood pressure in treated hypertensive individuals with the Mthfr 677tt genotype is responsive to intervention with riboflavin: findings of a targeted randomized trial. Hypertension. (2013) 61:1302–8. 10.1161/HYPERTENSIONAHA.111.01047 23608654

[67] McNulty H. Dowey le RC, Strain JJ, Dunne A, Ward M, Molloy AM, et al. Riboflavin lowers homocysteine in individuals homozygous for the Mthfr 677c->T polymorphism. Circulation. (2006) 113:74–80. 10.1161/CIRCULATIONAHA.105.580332 16380544

[68] Wang TJ Zhang F Richards JB Kestenbaum B van Meurs JB Berry D. Common genetic determinants of vitamin d insufficiency: a genome-wide association study. Lancet. (2010) 376:180–8. 10.1016/S0140-6736(10)60588-0 20541252 PMC3086761

[69] Nimitphong H Saetung S Chanprasertyotin S Chailurkit LO Ongphiphadhanakul B. Changes in circulating 25-Hydroxyvitamin D according to Vitamin D binding protein genotypes after vitamin D(3) or D(2)supplementation. Nutr J. (2013) 12:39. 10.1186/1475-2891-12-39 23556437 PMC3637219

[70] Rozmus D Plominski J Augustyn K Cieslinska A. Rs7041 and Rs4588 Polymorphisms in vitamin D binding protein gene (Vdbp) and the risk of diseases. Int J Mol Sci. (2022) 23:933. 10.3390/ijms23020933 35055118 PMC8779119

[71] Rozmus D Ciesielska A Plominski J Grzybowski R Fiedorowicz E Kordulewska N. Vitamin D binding protein (Vdbp) and its gene polymorphisms-the risk of malignant tumors and other diseases. Int J Mol Sci. (2020) 21:7822. 10.3390/ijms21217822 33105665 PMC7659952

[72] Karras SN Dursun E Alaylioglu M Gezen-Ak D Annweiler C Al Anouti F. Investigating the role of functional polymorphism of maternal and neonatal Vitamin D binding protein in the context of 25-Hydroxyvitamin D cutoffs as determinants of maternal-neonatal Vitamin D status profiles in a sunny mediterranean region. Nutrients. (2021) 13:3082. 10.3390/nu13093082 34578960 PMC8467735

[73] Nissen J Vogel U Ravn-Haren G Andersen EW Madsen KH Nexo BA. Common variants in Cyp2r1 and Gc genes are both determinants of serum 25-hydroxyvitamin d concentrations after uvb irradiation and after consumption of vitamin D(3)-fortified bread and milk during winter in denmark. Am J Clin Nutr. (2015) 101:218–27. 10.3945/ajcn.114.092148 25527766

[74] Rahman MM Hosen MB Faruk MO Hasan MM Kabir Y Howlader MZH. Association of vitamin D and vitamin D binding protein (Dbp) gene polymorphism with susceptibility of type 2 diabetes mellitus in Bangladesh. Gene. (2017) 636:42–7. 10.1016/j.gene.2017.09.008 28888576

[75] Yao P Sun L Lu L Ding H Chen X Tang L. Effects of genetic and nongenetic factors on total and bioavailable 25(Oh)D responses to vitamin D supplementation. J Clin Endocrinol Metab. (2017) 102:100–10. 10.1210/jc.2016-2930 27768857

[76] Lieu PT Heiskala M Peterson PA Yang Y. The roles of iron in health and disease. Mol Aspects Med. (2001) 22:1–87. 10.1016/s0098-2997(00)00006-6 11207374

[77] Dewi Shinta Asmarinah Chris Adhiyanto Min. The association of TMPRSS6 gene polymorphism and iron intake with iron status among under-2-year-old children in Lombok, Indonesia. Nutrients. (2019). Apr 19;11(4):878. 10.3390/nu11040878 31010126 PMC6521251

[78] Pei SN Ma MC You HL Fu HC Kuo CY Rau KM. Tmprss6 Rs855791 polymorphism influences the susceptibility to iron deficiency anemia in women at reproductive age. Int J Med Sci. (2014) 11:614–9. 10.7150/ijms.8582 24782651 PMC4003547

[79] Al-Amer O Hawasawi Y Oyouni AAA Alshehri M Alasmari A Alzahrani O. Study the association of transmembrane serine protease 6 gene polymorphisms with iron deficiency status in Saudi Arabia. Gene. (2020) 751:144767. 10.1016/j.gene.2020.144767 32422234

[80] Gan W Guan Y Wu Q An P Zhu J Lu L. Association of Tmprss6 Polymorphisms with ferritin, hemoglobin, and type 2 diabetes risk in a Chinese Han population. Am J Clin Nutr. (2012) 95:626–32. 10.3945/ajcn.111.025684 22301935

[81] De Falco L Tortora R Imperatore N Bruno M Capasso M Girelli D. The Role of Tmprss6 and Hfe variants in iron deficiency anemia in celiac disease. Am J Hematol. (2018) 93:383–93. 10.1002/ajh.24991 29194702

[82] Parys JB Bultynck G. Calcium signaling in health, disease, and therapy. Biochim Biophys Acta Mol Cell Res. (2018) 1865(11 Pt B):1657–9. 10.1016/j.bbamcr.2018.08.019 30798945

[83] Tankeu AT Ndip Agbor V Noubiap JJ. Calcium supplementation and cardiovascular risk: a rising concern. J Clin Hypertens (Greenwich). (2017) 19:640–6. 10.1111/jch.13010 28466573 PMC8030811

[84] Chiodini I Bolland MJ. Calcium supplementation in osteoporosis: useful or harmful? Eur J Endocrinol. (2018) 178:D13–25. 10.1530/EJE-18-0113 29440373

[85] He J Dong H Liu J Yang X Guo Y Yang S. Important roles of the Ca 2+ -sensing receptor in vascular health and disease. Life Sci. (2018) 209:217–27. 10.1016/j.lfs.2018.08.016 30098342

[86] O'Seaghdha CM Yang Q Glazer NL Leak TS Dehghan A Smith AV. Common variants in the calcium-sensing receptor gene are associated with total serum calcium levels. Hum Mol Genet. (2010) 19:4296–303. 10.1093/hmg/ddq342 20705733 PMC2951868

[87] Kapur K Johnson T Beckmann ND Sehmi J Tanaka T Kutalik Z. Genome-wide meta-analysis for serum calcium identifies significantly associated snps near the calcium-sensing receptor (Casr) gene. PLoS Genet. (2010) 6:e1001035. 10.1371/journal.pgen.1001035 20661308 PMC2908705

[88] Lewis CM Vassos E. Polygenic risk scores: from research tools to clinical instruments. Genome Med. (2020) 12:44. 10.1186/s13073-020-00742-5 32423490 PMC7236300

[89] Qi Q Chu AY Kang JH Jensen MK Curhan GC Pasquale LR et al. Sugar-sweetened beverages and genetic risk of obesity. N Engl J Med. (2012) 367:1387–96. 10.1056/NEJMc1213563 22998338 PMC3518794

[90] Kan J Wu F Wang F Zheng J Cheng J Li Y. Phytonutrients: sources, bioavailability, interaction with gut microbiota, and their impacts on human health. Front Nutr. (2022) 9:960309. 10.3389/fnut.2022.960309 36051901 PMC9424995

[91] Kaprio J. Twins and the mystery of missing heritability: the contribution of gene-environment interactions. J Intern Med. (2012) 272:440–8. 10.1111/j.1365-2796.2012.02587.x 22934540 PMC4422871

[92] Morgenstern JD Rosella LC Costa AP Desouza RJ Anderson LN. Perspective: big data and machine learning could help advance nutritional epidemiology. Adv Nutri. (2021) 12:621–31. 10.1093/advances/nmaa183 33606879 PMC8166570

[93] Lin E Lane HY. Machine learning and systems genomics approaches for multi-omics data. Biomark Res. (2017) 5:2. 10.1186/s40364-017-0082-y 28127429 PMC5251341

[94] Colmenar JM Winkler SM Kronberger G Maqueda E Hidalgo JI. Predicting glycemia in diabetic patients by evolutionary computation and continuous glucose monitoring. ACM. (2016) 3:1393–400. 10.1145/2908961.2931734

[95] Montaez CAC Fergus P Montaez AC Hussain A Chalmers C. Deep Learning Classification of Polygenic Obesity using Genome Wide Association Study SNPs. In: 2018 International Joint Conference on Neural Networks (Rio de Janeiro: IJCNN) (2018).

[96] Zhang SJ Meng P Zhang J Jia P Lin J Wang X. Machine learning models for genetic risk assessment of infants with non-syndromic orofacial cleft. Genom Proteom Bioinf. (2018) 16:354–64. 10.1016/j.gpb.2018.07.005 30578914 PMC6364041

[97] Kan J Li A Zou H Chen L Du J A. Machine learning based dose prediction of lutein supplements for individuals with eye fatigue. Front Nutr. (2020) 7:577923. 10.3389/fnut.2020.577923 33304916 PMC7691662

[98] Eldridge AL Piernas C Illner AK Gibney MJ Gurinovic MA de Vries JHM. Evaluation of new technology-based tools for dietary intake assessment-an Ilsi Europe dietary intake and exposure task force evaluation. Nutrients. (2018) 11:55. 10.3390/nu11010055 30597864 PMC6356426

